# Expansion effect of romiplostim on hematopoietic stem and progenitor cells versus thrombopoietin and eltrombopag

**DOI:** 10.1007/s12185-024-03853-6

**Published:** 2024-09-20

**Authors:** Yuta Tezuka, Naoki Onoda, Tatsuya Morishima, Yoshiki Sumitomo, Keigo Nishii, Hitoshi Takizawa, Masayuki Kai

**Affiliations:** 1 Research Division, Research Unit, Biomedical Science Research Laboratories 2, Kyowa Kirin Co., Ltd, 3-6-6, Asahi-Machi, Machida-Shi, Tokyo, 194-8533 Japan; 2 Research Core Function Laboratories, Research Unit, Research Division, Kyowa Kirin Co., Ltd, Tokyo, Japan; 3 Laboratory of Stem Cell Stress, International Research Center for Medical Sciences (IRCMS), Kumamoto University, Kumamoto, Japan; 4 Laboratory of Hematopoietic Stem Cell Engineering, IRCMS, Kumamoto University, Kumamoto, Japan; 5 Center for Metabolic Regulation of Healthy Aging (CMHA), Kumamoto University, Kumamoto, Japan

**Keywords:** HSPC, Romiplostim, TPO

## Abstract

Romiplostim, a thrombopoietin (TPO) receptor agonist, is a clinically approved drug that is clearly effective in reconstituting hematopoiesis in refractory aplastic anemia and　idiopathic thrombocytopenic purpura. However, the mechanism underlying its biological effect is unknown, and its differences from other TPO receptor agonists remain unclear. Therefore, we determined the in vitro expansion effect of romiplostim on human CD34 + hematopoietic stem and progenitor cells (HSPCs) versus recombinant human TPO (rhTPO) and another clinically available drug, eltrombopag. We also performed single-cell RNA-seq to determine effects of romiplostim on CD34 + HSPCs at the molecular level. The maximum expansion effect of romiplostim on total CD34 + cells, CD34 + CD38 + progenitor cells, and CD34 + CD38 − immature cells was comparable to that of rhTPO, but higher than that of eltrombopag, particularly on CD34 + CD38 − immature cells. Single-cell RNA-seq analysis revealed that both romiplostim and eltrombopag induced signatures driven by rhTPO, but romiplostim induced molecular changes related to RHOA signaling in the most primitive HSPC subsets that were partially driven or not driven by eltrombopag. Additionally, romiplostim did not induce *TFRC* expression as was observed with eltrombopag. In conclusion, romiplostim expands and affects human HSPCs similar to rhTPO, but partially different from eltrombopag in terms of induction of gene expression.

**Supplementary Information:**

The online version contains supplementary material available at 10.1007/s12185-024-03853-6.

## Introduction

Hematopoietic stem and progenitor cells (HSPCs) are a heterogeneous population comprising hematopoietic stem cells (HSCs), multipotent progenitor cells (MPPs), and lineage-committed progenitor cells, which develop and differentiate into specific types of blood lineages, including platelets, red blood cells, and lymphocytes. Defects in the development of any of the populations within HSPCs can lead to hematological disorders. Aplastic anemia (AA) is a condition involving bone marrow failure characterized by complex symptoms of thrombocytopenia, anemia, and neutropenia [1]. AA is primarily mediated by T-cell autoimmunity, wherein cytotoxic T cells attack HSPCs, resulting in cytopenia [1, 2]. Therefore, apart from blood transfusion, suppression of T cells and/or expansion, reconstitution, and maintenance of HSPCs is the current therapeutic strategy for the recovery of homeostatic hematopoiesis in AA.

Thrombopoietin (TPO) is a multifunctional hematopoietic growth factor that stimulates cellular function by binding to the TPO receptor (TPOR) and, consequently, triggering intracellular signaling. TPOR is expressed on HSCs, megakaryocyte progenitors, megakaryocytes, and platelets and regulates stem cell function and megakaryopoiesis [3, 4]. TPO activates downstream signaling including Janus kinase/signal transducers and activators of transcription (JAK/STAT), mitogen-activated protein kinase (MAPK), and phosphoinositide 3-kinase/AKT (PI3K/AKT), thereby participating in the expansion and maturation of megakaryocytes [5]. Moreover, studies using TPO − / − mice have suggested that TPO regulates the expansion and maintenance of HSCs [6].

Considering that TPO–TPOR activates the downstream signaling pathways involved in hematopoiesis, TPOR agonists have been developed for the treatment of hematologic disorders, including AA. Romiplostim, a TPO-mimetic peptide fused to a human IgG1 Fc, has been approved for the treatment of idiopathic thrombocytopenic purpura in the United States, Japan, and other countries worldwide and for the treatment of AA in Asian countries, including Japan [7]. In clinical studies of patients with refractory AA, romiplostim produced durable trilineage responses, demonstrating its efficacy in hematopoiesis recovery [8].

Despite achieving outstanding outcomes with romiplostim treatment in the clinical setting, its mechanisms of action with regard to hematopoietic recovery in refractory AA are unknown. Moreover, the difference in its mechanism of action with other TPOR agonists remains unclear. Romiplostim can promote megakaryocytic colony formation from murine bone marrow cells and stimulate the proliferation of human TPOR-expressing cell lines in vitro, which partially explains its mechanism of action in platelet development [9]. In a phase 2 clinical study, patients with refractory AA who were previously treated with immunosuppressive therapy received romiplostim during the study period, and responders had a higher concentration of CD34 + CD38 − cells and colony-forming blood progenitors in the bone marrow at week 53 than that at baseline; this suggests that romiplostim may demonstrate an expansion effect on both primitive subsets and progenitors within HSPCs [10]. However, investigational studies of the expansion effects of romiplostim on human HSPCs are limited. Therefore, to characterize and understand the biological effects of romiplostim on hematopoiesis in humans, we studied the in vitro effects of romiplostim on human HSPCs and compared them with those of the recombinant human TPO (rhTPO) and another TPOR agonist—eltrombopag—which is an orally administered small molecule also approved for the clinical treatment of hematological disorders, including AA [11]. The effects of rhTPO and eltrombopag on human HSPCs have been well characterized, which will serve as a benchmark in this study. We investigated the ability of romiplostim to expand HSPCs, as the expansion of these subsets is a key step in the reconstitution of hematopoiesis.

## Materials and methods

### *Proliferation assay of CD34* + *cells*

Frozen human cord blood (CB)–derived CD34 + cells (Lonza, Basel, Switzerland) were thawed and seeded at a density of 2 × 10^5^/mL in StemSpan™ Serum-Free Medium II (STEMCELL Technologies, Vancouver, Canada). Romiplostim (Kyowa Kirin Co., Ltd., Tokyo, Japan), eltrombopag (ChemScene, New Jersey, USA), recombinant human TPO (rhTPO; PeproTech, New Jersey, USA), or the vehicle dimethyl sulfoxide (DMSO) were added to the cells, after which the cells were incubated for 7 days at 37℃ and 5% CO_2_. Cells were stimulated with romiplostim at different concentrations ranging from 0.01 to 1000 ng/mL, which covers the maximum drug concentration (C_max_) value in patients with refractory AA treated with 10 µg/kg romiplostim once weekly for 4 weeks (median: 4.71 ng/mL) [8]. Eltrombopag was tested at a concentration range of 0.1–10,000 ng/mL, which covers the C_max_ value in patients with AA treated with 25 mg of eltrombopag (6.41 ± 4.20 µg/mL) [12]. rhTPO was stimulated at a concentration of 10 ng/mL, which is above serum levels in patients with AA and healthy donors (2728 ± 1074 pg/mL and 95.3 ± 54.0 pg/mL, respectively) and is sufficient as a positive control for the current study [13]. In some experiments, 5 ng/mL stem cell factor (SCF; PeproTech) and 5 ng/mL FMS-like tyrosine kinase 3 ligand (FLT3L; Miltenyi Biotec, Bergisch Gladbach, Germany) were added with the stimulants. All samples contained 0.68% DMSO. Flow cytometry was performed on the day of analysis. Cells were stained with anti-human CD34-APC (BD Biosciences, New Jersey, USA), anti-human CD38-PE/Cy7 (BD Pharmingen, New Jersey, USA), and anti-human Lineage Cocktail-Brilliant Violet 510 (BioLegend, California, USA) to determine CD34 + CD38 + and CD34 + CD38 − cells within the lineage marker–negative fraction. Dead cells were excluded by staining with 7-AAD (BD Pharmingen). CountBright Absolute Counting Beads (Invitrogen, Massachusetts, USA) were mixed with each sample to determine the absolute count of cells. Cells were analyzed on BD FACSymphony™ A3 (BD Pharmingen), and the collected data were analyzed using BD FlowJo™ Software version 10.8.1 (BD Pharmingen). The absolute number of cells per total assay volume (cells/µL) was calculated by comparing the data with the results of the Counting Beads event.

### Single-cell RNA-seq

#### Sample preparation

Four batches of frozen human CB–derived CD34 + cells were thawed and mixed to prepare sufficient amount of cells. These cells were seeded at a density of 2 × 10^5^ cells/mL in StemSpanTM Serum-Free Medium II. TPOR agonists were added to the cells, after which the cells were incubated for 2 days at 37℃ and 5% CO_2_.

#### Library preparation

For each sample, the quantity and viability of cells were confirmed, and the absence of aggregated cells or debris was microscopically observed. Single cells were encapsulated into emulsion droplets using Chromium Connect (10 × Genomics). Single-cell RNA-seq libraries were constructed using Chromium Next GEM Automated Single Cell 3′ Library and Gel Bead Kit v. 3.1 (10 × Genomics), Chromium Next GEM Automated Chip G Single Cell Kit (10 × Genomics), and Dual Index Kit TT Set A (10 × Genomics) following the manufacturer’s instructions (CG000286_Rev_E). The libraries were sequenced using Illumina NextSeq 2000.

#### Data processing

Sequencing results (BCL files) were converted into FASTQ files and mapped to the human reference genome (GRCh38) using Cell Ranger software (v. 6.0.0; 10 × Genomics). Downstream analysis was performed using the R package, Seurat (v. 3.2.2). The output matrices from Cell Ranger were converted into Seurat objects, and quality control of the obtained data was performed to retain the cells with more than 200 genes and fewer than 15% of the mitochondrial reads. The objects from each sample were preprocessed using the Seurat standard workflow with the functions of NormalizeData and FindVariableFeatures; subsequently, the outputs were integrated by the Seurat anchor-based integration workflow [14]. To remove unwanted sources of variation derived from the cell cycle phase or sex-based differences among the cells, the difference between G2M and S phase scores for the cell cycle phase and the expression levels of *XIST* and *RPS4Y1*, which have been reported as sex biomarkers in the transcriptome [15], were regressed out when the data were scaled with the ScaleData function. G2M and S phase scores were calculated using the CellCycleScoring function [16]. Subsequently, the scaled data were used for principal component analysis, dimensionality reduction, and unsupervised clustering. For further analysis, differentially expressed genes (DEGs) were obtained using the FindMarkers function with the “test” option as “negbinom”. The score for TPO stimulation was also calculated using the AddModuleScore function with the gene sets reportedly increased or decreased in HPC-7 cells after stimulation by murine recombinant TPO [17]. Trajectory and pseudotime analyses were performed using the R package, Monocle3 (v. 1.0.0), following its standard workflow [18–20]. All graphic figures were created using R version 3.6.1.

#### Gene ontology enrichment and pathway analyses

Gene ontology (GO) enrichment analysis was performed using Metascape [21] with default parameters. The canonical pathway, upstream regulator, and biological function analyses were generated using QIAGEN IPA (QIAGEN Inc., Venlo, The Netherlands, https://digitalinsights.qiagen.com/IPA) [22].

## Results

### Romiplostim induces expansion of CD34 + cells in vitro

**Fig. 1:**
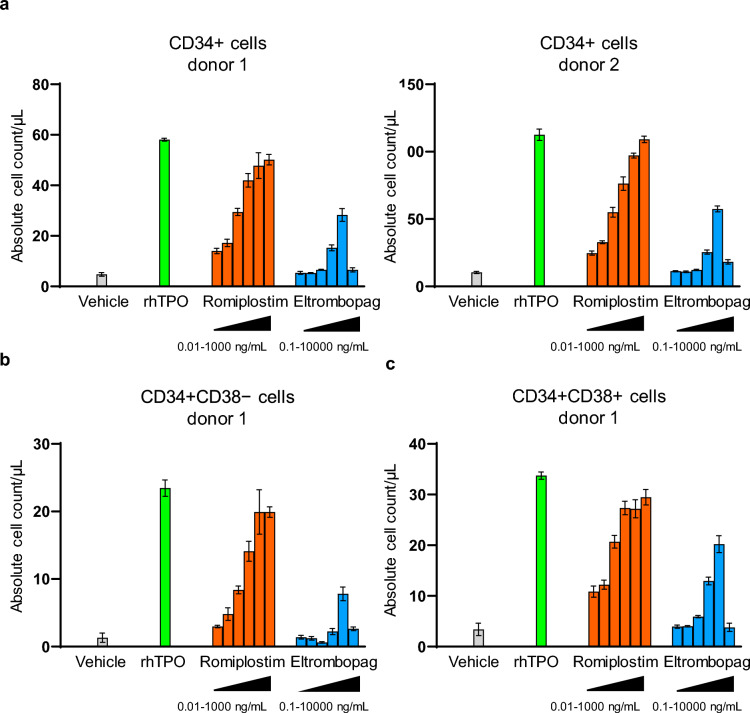
Romiplostim induces expansion of CD34 + cells. (**a, b, c**) Human cord blood–derived CD34 + cells were treated with romiplostim (0.01, 0.1, 1, 10, 100, or 1000 ng/mL), eltrombopag (0.1, 1, 10, 100, 1000, or 10,000 ng/mL), recombinant human thrombopoietin (10 ng/mL), or vehicle (DMSO) for 7 days. The cells were then collected and counted by flow cytometry. Absolute cell count per assay volume (cells/µL) of total CD34 + cells (**a**), CD34 + CD38 − cells (**b**), and CD34 + CD38 + cells (**c**) are shown. (**a**) Two different results of the same experiment performed using two different donor cells. Data are shown as mean ± standard error of the mean, n = 3. (**b**) and (**c**) Results obtained using CD34 + cells from donor 1

We examined the expansion effects of romiplostim, rhTPO, and eltrombopag on HSPCs. We used CD34 + cells derived from human CB, as HSPCs are highly enriched in CB. The results showed that all three TPOR agonists induced expansion of CD34 + cells when compared with the vehicle, and a similar result was obtained in samples collected from different donors (Fig. 1a, also see Fig. S1a for the gating strategy). Romiplostim demonstrated a concentration-dependent increase in CD34 + cell count. In contrast, eltrombopag demonstrated a concentration-dependent effect (up to 1000 ng/mL), but the CD34 + cell count declined at a concentration of 10,000 ng/mL. The maximum efficacy of romiplostim was similar to that of rhTPO but higher than the maximum efficacy of eltrombopag. Next, we examined which subset within the CD34 + cell population was affected by each stimulation. The more primitive cells were defined as CD34 + CD38 − , and the more differentiated cells were defined as CD34 + CD38 + (Fig. S1b). The absolute count of CD34 + CD38 − cells at maximum efficacy was different between stimulants, with romiplostim demonstrating higher activity than eltrombopag (Fig. 1b). The absolute count of CD34 + CD38 + cells at maximum efficacy was higher with romiplostim than with eltrombopag, but this difference between the two stimulants was smaller in CD34 + CD38 + cells than that in CD34 + CD38 − cells (Fig. 1c). The maximum effect of romiplostim reached the effect of rhTPO in both subsets (Fig. 1b and Fig. 1c). Interestingly, the expansion effect of eltrombopag at 10,000 ng/mL was lower than that at a lower concentration (1000 ng/mL) in both CD34 + CD38 − cells (Fig. 1b) and CD34 + CD38 + cells (Fig. 1c). Similar results were obtained with the culture condition containing SCF and FLT3L, which are the commonly known cytokines for expanding HSPCs (Fig. S2) [23]. Altogether, these results suggest that romiplostim induces expansion of HSPCs, including the primitive subsets, in a manner similar to that of rhTPO and at higher efficacy than that of eltrombopag in vitro.

### Romiplostim affects the megakaryocytic trajectory in HSPCs similar to rhTPO and eltrombopag

HSPCs are heterogeneous cells comprising HSCs, MPPs, and lineage-committed progenitor cells. Given that we had observed the expansion effects of romiplostim on both CD34 + CD38 − and CD34 + CD38 + subsets, we next attempted to identify which subset was affected by romiplostim and to examine whether the effects of romiplostim at the molecular level was similar to those of rhTPO and eltrombopag. For this, we performed a single-cell RNA-seq analysis. Human CB-derived CD34 + cells were stimulated with romiplostim, eltrombopag, rhTPO, or the vehicle DMSO in a serum-free suspension culture for 2 days and proceeded to single-cell RNA-seq analysis. As the expanding effects of romiplostim, rhTPO, and eltrombopag were similar whether or not SCF and FLT3L were included in the culture, the assay was done without SCF and FLT3L to observe the specific effects of these stimulants. This early time point was chosen to investigate the primary effect of stimulation and to avoid the effects secondary to proliferation. The tested concentration was selected at the point approximate to the C_max_ value or the serum concentration described earlier (10 ng/mL for romiplostim and rhTPO). Eltrombopag was tested at two concentrations covering the C_max_ value; at the concentration of 1 µg/mL, which demonstrated the maximum efficacy in expanding CD34 + cells, and at the concentration of 10 µg/mL, which demonstrated slight efficacy in expanding CD34 + CD38 − cells without expanding CD34 + CD38 + cells (Fig. 1).

**Fig. 2:**
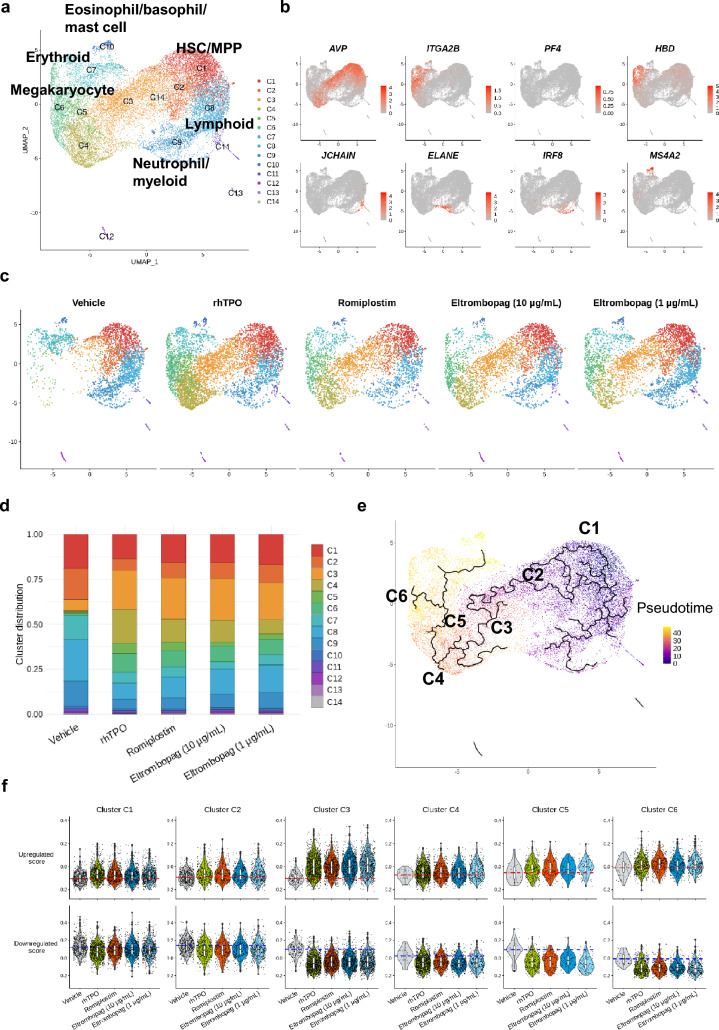
Romiplostim affects the megakaryocytic trajectory in HSPCs in a manner similar to that of recombinant human thrombopoietin (rhTPO) and eltrombopag. Single-cell RNA-seq was performed on human cord blood–derived CD34 + cells stimulated with thrombopoietin receptor agonists for 2 days in culture. (**a**) Uniform Manifold Approximation and Projection (UMAP) plot of unsupervised clustering of diversely expressed genes against the integrated data of all samples. Major blood cell populations were determined according to the transcriptome profiles and annotated to the corresponding cluster. (**b**) Expression of representative genes associated with hematopoietic stem cells (*AVP*), megakaryocytes (*ITGA2B* and *PF4*), eosinophils/basophils/mast cells (*MS4A2*), erythroids (*HBD*), myeloids (*IRF8*), lymphoids (*JCHAIN*), and neutrophils (*ELANE*) mapped on UMAP plot. (**c**) UMAP plot of each sample stimulated with either romiplostim, rhTPO, or eltrombopag at the indicated concentration or vehicle (DMSO). (**d**) Bar graph of cluster distribution for each sample. Cluster distribution was calculated as the relative value of one as a whole. (**e**) Pseudotime analysis was performed against the integrated data when cluster C1 was selected as the starting point. (**f**) Thrombopoietin activation score was generated and compared between samples on clusters C1 through C6. Upregulated score (top) and downregulated score (bottom) are shown individually. A dotted line is drawn on the mean value of the vehicle

Based on single-cell RNA-seq, 21,697 cells were collected and sequenced with adequate reads, that is, approximately hundred thousand or more per cell (Table S1). First, 14 clusters were obtained as a result of unsupervised clustering. The representative blood cell subsets were annotated, as they express specific genes, and were classified as follows: Cluster C6, megakaryocytes with relatively high expression of *ITGA2B* and *PF4*; C7, erythroid cells with *HBD* expression; C9, neutrophils/myeloid cells with expression of *IRF8* and *ELANE*; C10, eosinophils/basophils/mast cells with expression of *MS4A2*; and C11, lymphoid cells with expression of *JCHAIN* (Fig. 2a and 2b). Next, we classified cluster C1 as a population enriched in HSCs/MPPs, as they had relatively high expression of *AVP* (Fig. 2a and 2b). The rest of the clusters were presumed to be either progenitor-enriched cells or difficult-to-classify cells.

To characterize the effects of romiplostim on CD34 + cells, we first compared the results of romiplostim with the vehicle, rhTPO, and eltrombopag. Quantitative analysis for the proportion of each cluster (Fig. 2c and 2d) revealed that while the vehicle barely showed distribution at clusters C3, C4, C5, and C6, romiplostim stimulation induced remarkable expansion of these clusters, consistent with the results obtained with rhTPO and eltrombopag. Trajectory analysis revealed that cluster C1, marked as HSCs, proceeded to C6, annotated as megakaryocytes, through C2, C3, C4, and C5 (Fig. 2e) [24]. GO enrichment analysis of specifically expressed genes against clusters C1 through C6 showed that C4 and C5 were enriched for the cell cycle process (Fig. S3 and Table S2). Collectively, as romiplostim stimulation forced cells to distribute into clusters that differentiate into megakaryocytes and because these clusters were significantly cycling, we concluded that romiplostim induced proliferation of cells along the trajectory where HSCs differentiate into megakaryocytes. Moreover, a similar effect was induced by rhTPO and eltrombopag.

We next examined the similarity of rhTPO and romiplostim as well as eltrombopag in terms of TPOR signaling. Therefore, we generated a score that reflects the activation of TPOR signaling established by Comoglio et al. [17] and compared it between stimulants in clusters C1 through C6 individually. This score was calculated based on the gene expression levels of 23 genes with upregulated expression, including *Myc*, and downstream targets of JAK/STAT signaling, or 20 genes with downregulated expression, such as *Sox4* and *Hlf*, which are rapidly induced by recombinant mouse TPO stimulation. Indeed, rhTPO, compared with vehicle, induced upregulation of the upregulated score and downregulation of the downregulated score on every cluster investigated, assuring that this prediction score is effective in our system (Fig. 2f). The results showed that romiplostim and eltrombopag induced qualitative changes in scores in the same manner as rhTPO from clusters C1 through C6 (Fig. 2f). This suggests that romiplostim, along with eltrombopag, activates TPOR signaling along the megakaryocytic trajectory.

### Romiplostim affects the primitive subsets of HSPCs

**Fig. 3:**
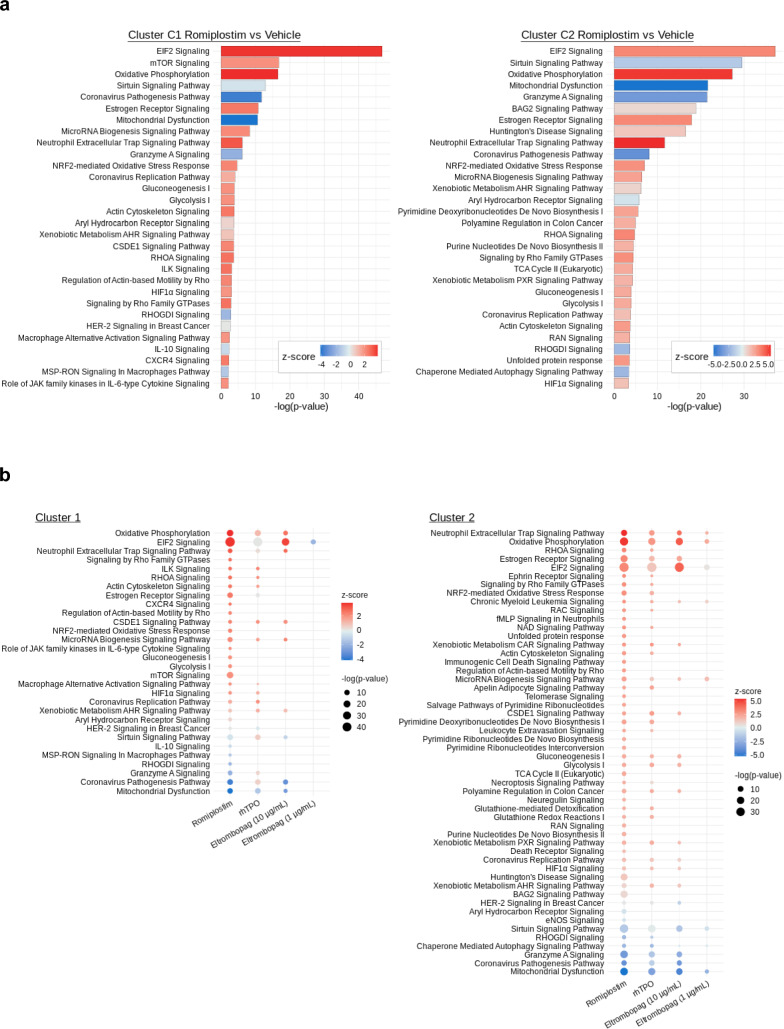
Romiplostim affects the primitive subsets of hematopoietic stem and progenitor cells (HSPCs). (**a**) Lists of pathways generated using differentially expressed genes (DEGs) at log fold-change of over 0.18 between romiplostim and vehicle (DMSO) against clusters C1 and C2. The pathways were filtered with − log10 (*p*-value) ≥ 2, and the top 30 pathways with the highest − log10 (*p*-value) are listed in the descending order. Pathways for which z-score was not determined were excluded. (**b**) Heat map of pathways compared between thrombopoietin receptor agonists. Pathways were generated as described above and listed in the order of z-score. Bubble size was associated with − log (*p*-value)

The durable recovery of hematopoiesis in AA is dependent on the expansion, self-maintenance, and differentiation of HSCs. Therefore, we then aimed to identify the specific effects of romiplostim and other TPOR agonists on the most primitive subsets of HSPCs in the single-cell RNA-seq analysis. Differentially expressed genes (DEGs) between each TPOR agonist and vehicle were generated, and pathway analysis was performed for clusters C1 and C2 individually (Tables S3 and S4). Figure 3a shows the lists of pathways generated using DEGs between romiplostim and vehicle. Figure 3b shows a heat map comparison of all pathways developed by each TPOR agonist. Eltrombopag at a concentration of 1 µg/mL in cluster C1 had a very low amount of DEGs to generate pathway lists (Table S3).

One of the top pathways significantly induced by romiplostim in clusters C1 and C2 was EIF2 signaling (*p*-value < 0.01), which is the key pathway for protein synthesis. Moreover, we found this signature to be significantly upregulated by rhTPO (*p*-value < 0.01) and 10 µg/mL eltrombopag (*p*-value < 0.01).

Another pathway significantly induced by romiplostim in both clusters C1 and C2 was oxidative phosphorylation (*p*-value < 0.01). Similar terms such as mitochondrial dysfunction and sirtuin signaling pathway were also listed. Additionally, rhTPO (*p*-value < 0.01) and eltrombopag (*p*-value < 0.01) also significantly induced these pathways.

Interestingly, some pathways were induced by romiplostim and rhTPO but not by eltrombopag. For instance, Ras homolog family member A (RHOA) signaling was listed as being significantly induced by romiplostim (*p*-value < 0.01) in both clusters C1 and C2 but not by 10 µg/mL eltrombopag (*p*-value = 0.37 and 0.61, respectively). The induction of RHOA signaling by rhTPO was significant in cluster C1 but not in C2 (*p*-value > 0.01 and = 0.02, respectively). Similar terms such as actin cytoskeleton signaling were also listed.

Taken together, while we observed similarities between romiplostim, rhTPO, and eltrombopag in terms of cluster distribution of HSPCs, as well as in TPO score along the megakaryocytic trajectory, the specific changes induced in the most primitive subsets were partially different.

### Eltrombopag at a high concentration induces *TFRC* expression not observed with romiplostim and rhTPO

**Fig. 4:**
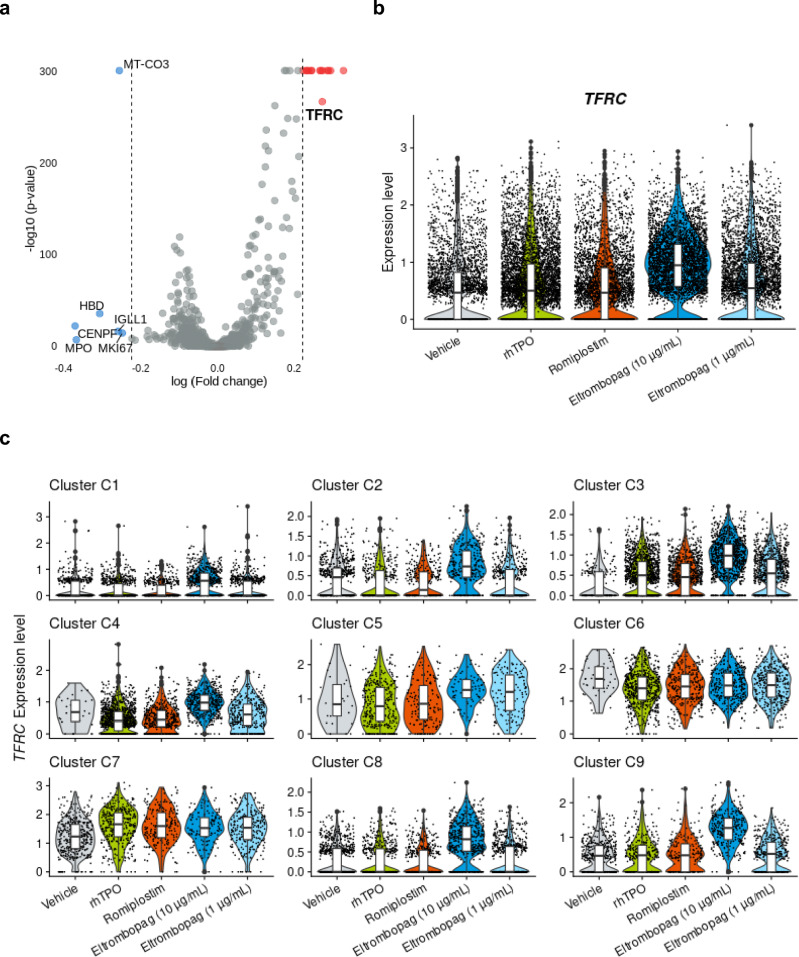
Eltrombopag at a concentration of 10 µg/mL has a unique effect. (**a**) Volcano plot of differentially expressed genes (DEGs) between eltrombopag 10 µg/mL and the integrated data of recombinant human thrombopoietin (rhTPO), romiplostim, and eltrombopag 1 µg/mL against total cells. The adjusted *p*-value was calculated based on bonferroni correction using all genes in the dataset. Dotted lines are drawn on fold change of 1.25. Red dots represent DEGs with higher expression on stimulation with eltrombopag 10 µg/mL, and blue dots represent DEGs with lower expression on stimulation with eltrombopag 10 µg/mL. (**b**, **c**) Gene expression of *TFRC* in each sample within total cells (**b**) or individually in clusters C1 through C9 (**c**)

While we observed the inhibition of CD34 + cell expansion with eltrombopag at 10 µg/mL in the proliferation assay (Fig. 1a), single-cell RNA-seq analysis demonstrated that this high concentration induced distribution and TPOR signaling on megakaryocytic trajectory, similarly to rhTPO and romiplostim. Based on this, we presumed that a unique characteristic of 10 µg/mL eltrombopag that is independent of TPOR may exist [25]. Therefore, to gain insights into the biology underlying the inhibitory effect, we searched for DEGs unique to 10 µg/mL eltrombopag. For this, we compared 10 µg/mL eltrombopag to the integrated data of rhTPO, romiplostim, and 1 µg/mL eltrombopag against total cells. The results showed that *TFRC*, which codes for transferrin receptor, was one of the genes with significantly higher expression (Adjusted *p*-value < 0.01) when stimulated with 10 μg/mL eltrombopag compared to the integrated data of other groups (Fig. 4a and Table S5)*.* A comparison between individual samples demonstrated that *TFRC* was uniquely induced by 10 µg/mL eltrombopag (Fig. 4b). Analysis against individual clusters also presented similar results (Fig. 4c).

Eltrombopag has an iron-chelating property, which presumably suggests that upregulated expression of *TFRC* is a phenotype for the iron demand [26]. Indeed, iron chelation can upregulate *TFRC* expression and inhibit cell proliferation [27, 28]. Consistent with this, *MKI67* expression, a proliferation marker, was relatively low when stimulated with 10 µg/mL eltrombopag (Fig. 4a). Therefore, high concentration of eltrombopag induces TPOR signaling and TPOR signaling-independent chelating activities, and the latter effect may be the cause of the inhibition of cell proliferation.

## Discussion

In the current study, we explored the expansion effects of romiplostim on human HSPCs versus rhTPO and eltrombopag. Romiplostim induced the expansion of HSPCs, including the subsets enriched in progenitor cells as well as primitive cells. The efficacy of romiplostim at a concentration close to the C_max_ value nearly reached that of rhTPO at concentration (10 ng/mL) exceeding the blood concentration, suggesting that romiplostim causes expansion of HSPCs at a clinically effective concentration. Single-cell RNA-seq analysis indicated that romiplostim preferentially induced cell distribution to the subsets along the megakaryocytic trajectory characterized as cycling cells at an early time point after stimulation. This suggests that the expanded CD34 + CD38 + progenitors observed in the culture assay consists of cells differentiating toward megakaryocytes. We also observed that romiplostim affected the expression of genes related to protein synthesis, mitochondrial regulation, and cytoskeleton rearrangement on the most primitive subsets of HSPCs.

Protein synthesis is a preceding step for cell proliferation. c-Myc is the pivotal transcription factor that couples translational activities to cell division [29]. During hematopoiesis, c-Myc primes HSCs for the differentiation and induction of the proliferation of progenitor cells [30]. In the present study, *MYC* expression was upregulated by romiplostim on primitive subsets (clusters C1 and C2), generating pathways related to protein synthesis. Moreover, cluster C3, representing relatively high expression of *MYC* and molecular functions related to protein processing, was induced by romiplostim. Therefore, romiplostim may initiate translation and related processes under the regulation of c-Myc in primitive subsets of HSPCs for division and differentiation into megakaryocytic progenitors.

Mitochondrial regulation of cell cycle and cell proliferation is another important effect during cell division or maintenance. The process of cell division creates a high demand for oxygen, thereby making the cells highly vulnerable to oxidative stress. Thus, an antioxidant stress response is essential for protecting cells from such threats, including HSCs, which need to be preserved and maintained throughout lifetime. TPO induces and regulates mitochondrial activity in mouse HSCs, which induces adaptability and resistance to apoptosis and oxidative stress [31]. As romiplostim induces the expression of genes related to antioxidant stress response in the most primitive subset of HSPCs, this drug may also play important roles in the maintenance of HSCs similar to TPO.

RHOA GTPase is a key component of intracellular signaling that regulates cytoskeleton rearrangement, adhesion, and migration when cells are stimulated by outer cues [32]. In pathway analysis, the expression of genes related to actin cytoskeleton signaling and RHOA signaling was detected in the most primitive subset of HSPCs stimulated with romiplostim. Among the genes, *ACTB* and *ATCG1* codes for proteins composing the filamentous actin [33]. *PFN1* coding for profilin and *CFL1* coding for cofilin are molecules tuning the elongation and shortening of the actin filaments [33]. All these genes are involved in the actin dynamics, regulated by RHOA signaling. Given that our in vitro liquid culture system is biased towards cells dividing rather than entering G0 phase or self-renewing, and that romiplostim expanded the primitive subsets, the dynamic actin remodeling signals induced by romiplostim are presumed to be an event occurring during the process of cell division. In addition, we also detected CXCR4 signaling, which was only significantly induced by romiplostim. The CXCL12/CXCR4 axis, through RHOA signaling, induces the mobilization of HSPCs, both from the bone marrow niche into the circulation and vice versa [34]. Therefore, it is interesting to study the phenotypes other than proliferation and the molecular mechanisms of romiplostim and eltrombopag associated with RHOA signaling.

Moreover, the important finding is that the effects of romiplostim on HSCs and cells of the megakaryocytic trajectory are quite similar to those of rhTPO. Consistently, romiplostim induced changes that were highly similar to those driven by rhTPO on HSPCs at the molecular level, as demonstrated through TPO score analysis and pathway analysis, confirming that the characteristics expressed by romiplostim are dependent on TPOR signaling.

In contrast, some differences were observed between romiplostim and eltrombopag. In studies on CD34 + cell expansion, eltrombopag demonstrated the maximum response at a concentration of 1 µg/mL, but the response decreased at a higher concentration (10 µg/mL), and the effect was higher with romiplostim. Additionally, although eltrombopag 1 µg/mL induced expansion of CD34 + CD38 + progenitors in a similar manner as romiplostim, the expansion effect of eltrombopag on CD34 + CD38 − immature cells was relatively poor. Based on these results, we tested both concentrations (eltrombopag 1 µg/mL and 10 µg/mL) for single-cell RNA-seq analysis and compared to the effects of romiplostim. Eltrombopag at both concentrations induced cluster distribution to the megakaryocytic trajectory as well as induction of TPOR signaling, as demonstrated through TPO score changes. This suggests that eltrombopag induces qualitative changes similar to romiplostim and rhTPO. However, the effect of eltrombopag on the immature clusters including clusters C1 and C2 was relatively weaker than that of romiplostim, suggested by the fewer number of DEGs and pathways generated by eltrombopag compared to romiplostim. In contrast, the number of DEGs and pathways generated by eltrombopag were similar to those by romiplostim on the downstream cluster C6. Therefore, eltrombopag may weakly affect the primitive cells when compared with its effect on the progenitor cells. This finding is consistent with the results that eltrombopag (1 µg/mL), when compared with romiplostim, induced the proliferation of CD34 + CD38 + progenitors in a similar manner but to a lower extent in CD34 + CD38 − primitive cells. Accordingly, the difference in induction intensity on TPOR signaling between romiplostim and eltrombopag (1 µg/mL) may explain the difference in the effect observed. A comparative analysis of the downstream signaling strengths of TPOR between romiplostim and eltrombopag may provide more insights into understanding the differences.

Meanwhile, eltrombopag at a concentration of 10 µg/mL, but not at 1 µg/mL, induced *TFRC* expression in most subsets within HSPCs, but this expression was not induced by romiplostim and rhTPO. Eltrombopag possesses an iron-chelating property, which, in turn, may upregulate *TFRC* expression, a phenotype necessitating iron demand [26]. Iron is an essential supplement for cell division. Therefore, the low response of HSPC expansion by eltrombopag at 10 µg/mL may be due to the iron-chelating property. Meanwhile, the iron-chelating property of eltrombopag is reported to stimulate self-renewal of HSCs [25]. Therefore, eltrombopag 10 µg/mL may exert positive effects on HSPCs, which could not be defined in our cell expansion system. The clinical significance of eltrombopag’s TPOR-independent effects that we observed in vitro needs to be interpreted with caution.

How romiplostim and eltrombopag stimulate hematopoiesis in the context of AA is still an open question. Physiologically, endogenous TPO exists in the bloodstream. Therefore, it is speculated that romiplostim and eltrombopag stimulate TPOR in the presence of TPO. Broudy et al. found that romiplostim competes with TPO for binding to TPOR, but the development of megakaryocytic colonies was additive with TPO [35]. On the other hand, eltrombopag binds to the transmembrane domain of TPOR, does not compete with TPO, and has additive effects with TPO on platelet production [36]. As our culture was conducted in the absence of rhTPO, it will be interesting to observe the difference of romiplostim and eltrombopag in the presence of rhTPO. Future studies investigating the expansion effect of romiplostim and eltrombopag in a more physiological condition and on CD34 + cells derived from AA patients are warranted.

In summary, this is the first study which investigated the expansion effect and induction of molecular changes of romiplostim on human CD34 + HSPCs versus rhTPO and eltrombopag. The results indicate that romiplostim induces expansion of human HSPCs, including the primitive subsets, in a manner similar to rhTPO. Expansion of HSPCs is a key step to the recovery of hematopoiesis in hematological disorders; therefore, this activity may, in part, explain the benefit of romiplostim treatment in patients with refractory AA. Moreover, the effects of romiplostim on the most primitive subsets in HSPCs resembled those of rhTPO, which is a central player for HSC maintenance. These functional aspects of romiplostim may also contribute to the recovery of hematopoiesis in hematological disorders, but further research reporting confirmatory findings is awaited. Additionally, romiplostim and eltrombopag showed different responses in terms of CD34 + HSPC expansion. The outcome may be partly explained by the difference in the TPOR signal intensity and the presence or absence of iron-chelating property. Therefore, the distinct mechanism of action of romiplostim compared to eltrombopag may lead to the difference in outcomes in patients with refractory AA, but what difference our findings will make for the treatment of AA requires further investigation.

## Supplementary Information

Below is the link to the electronic supplementary material.

Supplementary file1 (DOCX 621 KB)

Supplementary file2 Table S1 Cell counts for each sample on single cell RNA-seq analysis. Table S2 Gene ontology (GO) enrichment analysis of specifically expressed genes against clusters C1, C2, C3, C4, C5, and C6. Table S3 List of differentially expressed genes generated by comparing each thrombopoietin receptor agonists and vehicle on clusters C1 and C2. Table S4 Pathway lists generated using differentially expressed genes on Supplementary Table S3. Table S5 List of differentially expressed genes generated by comparing 10 µg/mL eltrombopag to integrated data of recombinant human thrombopoietin, romiplostim, and 1 µg/mL eltrombopag against total cells (XLSX 1009 KB)

## Data Availability

The datasets generated during and/or analyzed during the current study are available in the NCBI GEO repository, GSE253936.
