# Supplementary material for: Expansion effect of romiplostim on hematopoietic stem and progenitor cells versus thrombopoietin and eltrombopag
Source: Int J Hematol. 2024 Sep 20;120(5):575–86. doi: 10.1007/s12185-024-03853-6 (PMC11513719; doi:10.1007/s12185-024-03853-6)
Supplement: Supplementary file 1 — Supplementary file1 (DOCX 621 KB) [file 12185_2024_3853_MOESM1_ESM.docx]

**Supplementary Materials**

**a**

**
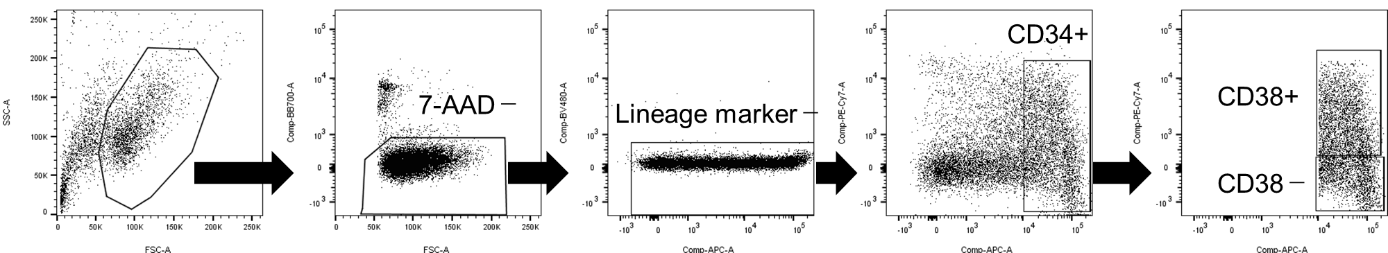
**

**b**

**
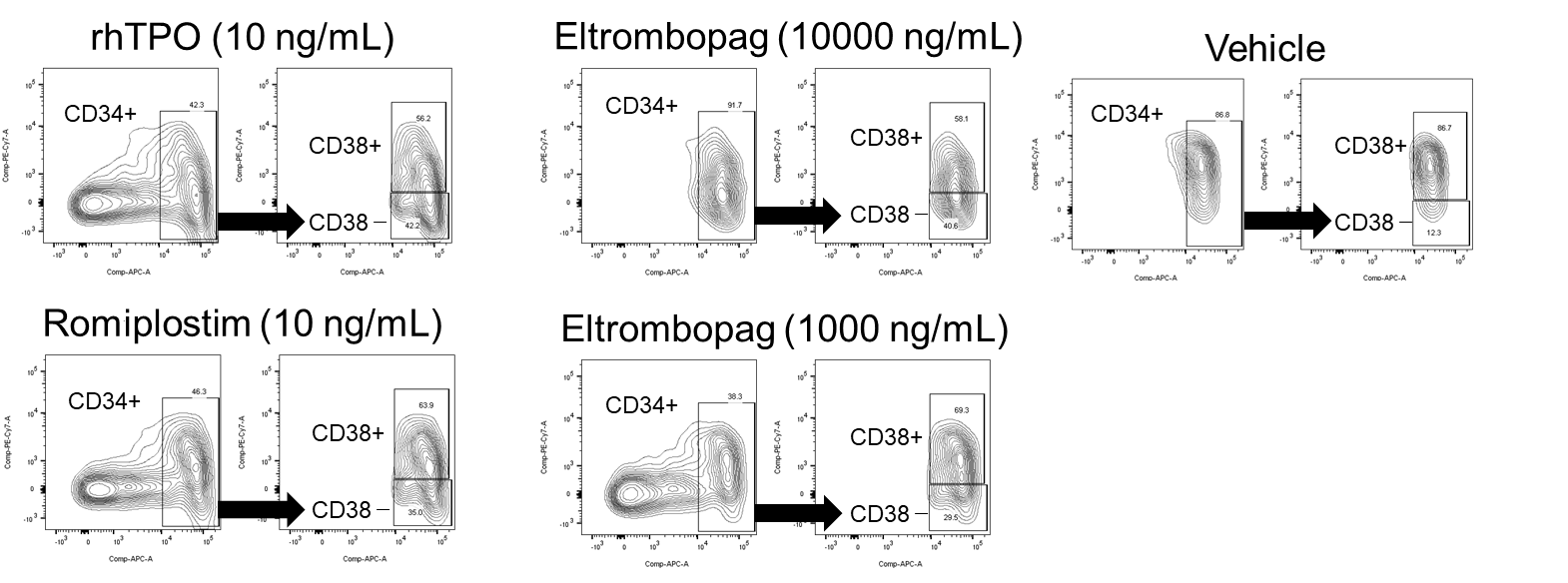
**

**Fig. S1** Representative flow cytometry plot data related to figure 1. **(a)**  Gating strategy for detecting CD34+ cells, CD34+CD38− cells, and CD34+CD38+ cells are shown. **(b)**  Representative plot data of CD34+ cells, CD34+CD38− cells, and CD34+CD38+ for recombinant human thrombopoietin (10 ng/mL), romiplostim (10 ng/mL), eltrombopag (10000 and 1000 ng/mL) and vehicle.

**
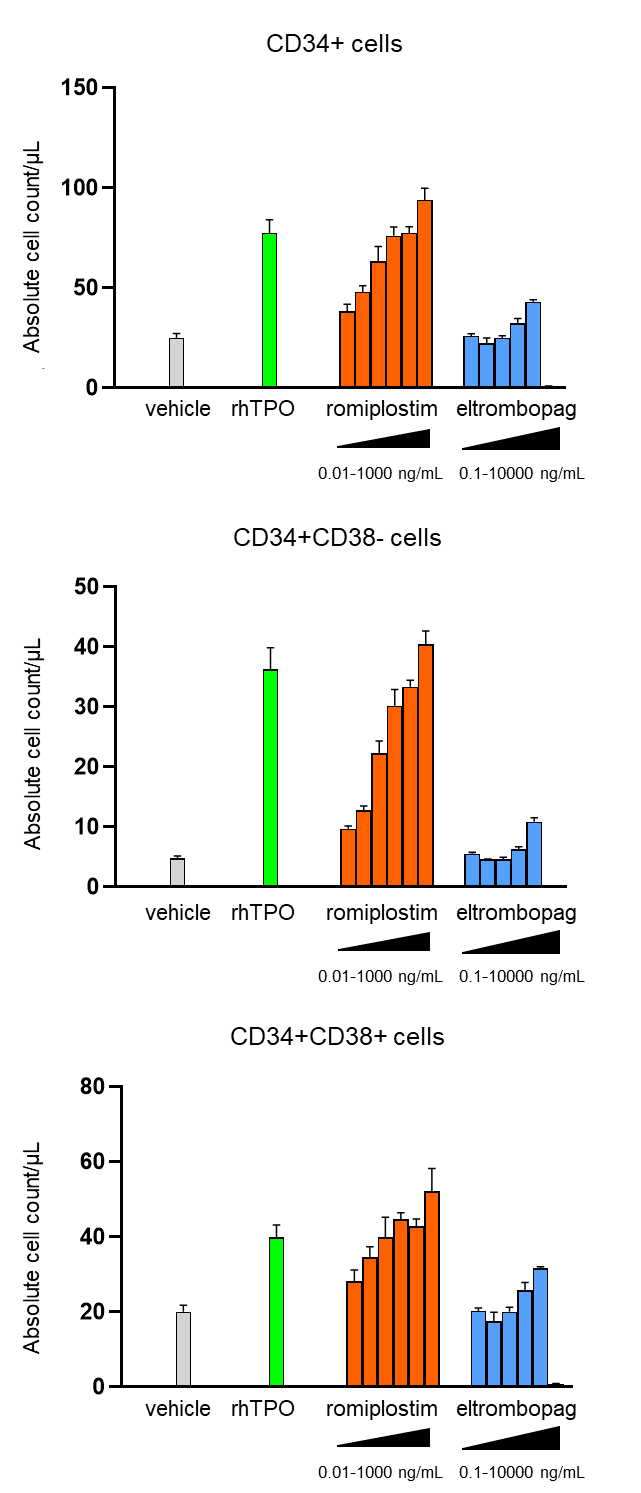
**

**Fig. S2** Romiplostim induces expansion of CD34+ cells in the presence of SCF and FLT3L. Human cord blood–derived CD34+ cells were treated with romiplostim (0.01, 0.1, 1, 10, 100, or 1000 ng/mL), eltrombopag (0.1, 1, 10, 100, 1000, or 10000 ng/mL), recombinant human thrombopoietin (10 ng/mL), or vehicle (DMSO) with SCF and Flt3l for 7 days The cells were then collected and counted by flow cytometry. Absolute ell counts per assay volume (cells/µL) of total CD34+ cells, CD34+CD38− cells, and CD34+CD38+ cells are shown. Data are shown as mean ± standard error of the mean, n=3


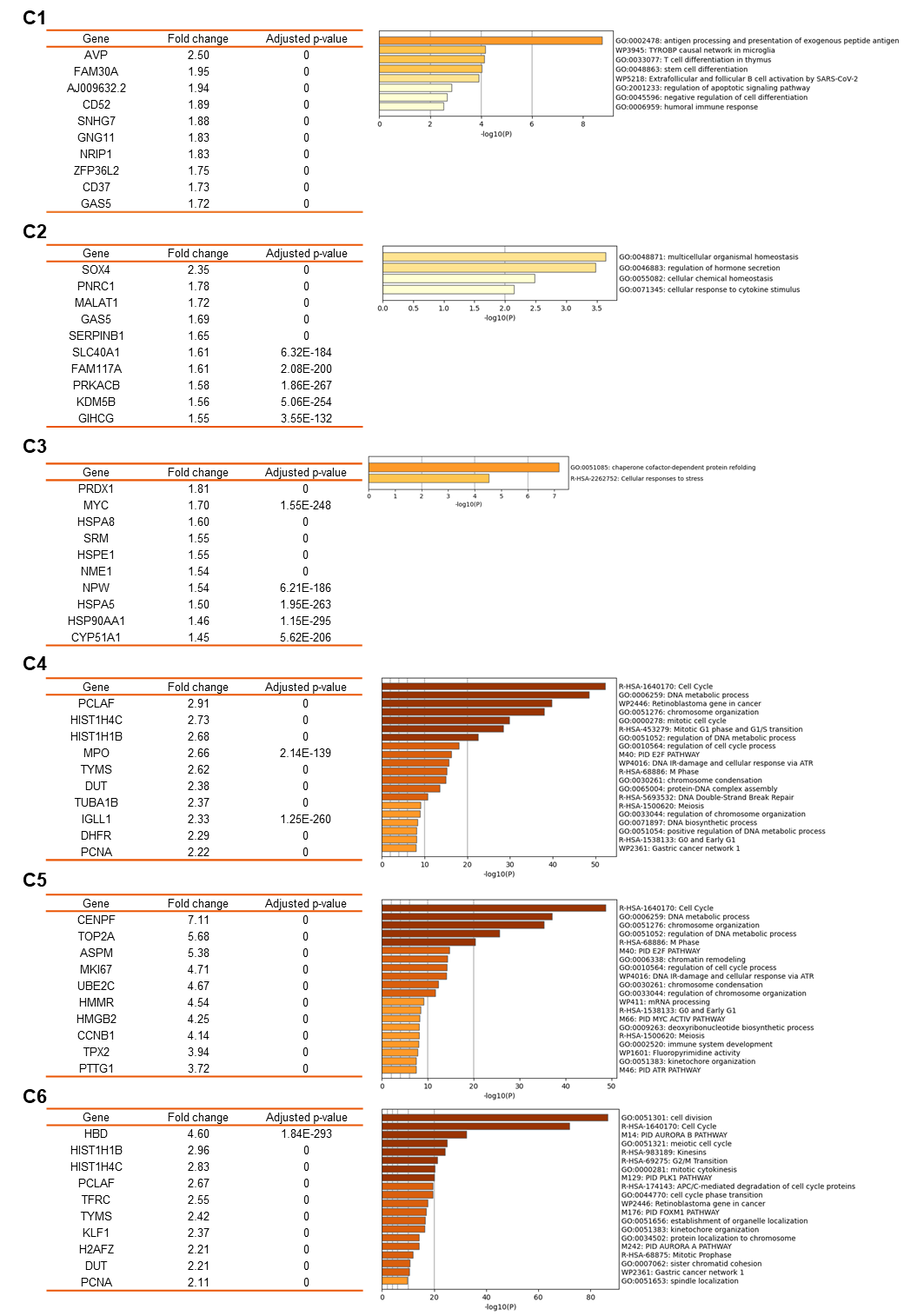


**Fig. S3** Gene ontology (GO) enrichment analysis against clusters C1, C2, C3, C4, C5, and C6. The top 10 genes with the highest fold change and a Bonferroni’s adjusted p-value of ≤0.05 in each cluster compared with total cells are shown. GO enrichment analysis against each cluster was performed. The top significantly enriched GO terms of the target genes in the cellular components, molecular functions, and biological processes are listed in the order of −log10 (p-value)
